# Verbascoside Protects Mice From Clostridial Gas Gangrene by Inhibiting the Activity of Alpha Toxin and Perfringolysin O

**DOI:** 10.3389/fmicb.2020.01504

**Published:** 2020-07-14

**Authors:** Jian Zhang, Shui Liu, Lining Xia, Zhongmei Wen, Naiyu Hu, Tingting Wang, Xuming Deng, Jiakang He, Jianfeng Wang

**Affiliations:** ^1^Department of Respiratory Medicine, The First Hospital of Jilin University, Jilin University, Changchun, China; ^2^Key Laboratory of Zoonosis Research, Ministry of Education, Institute of Zoonosis, College of Veterinary Medicine, Jilin University, Changchun, China; ^3^College of Veterinary Medicine, Xinjiang Agricultural University, Ürümqi, China; ^4^College of Animal Science and Technology, Guangxi University, Nanning, China

**Keywords:** alpha toxin, anti-infection, *Clostridium perfringens*, gas gangrene, perfringolysin O, verbascoside

## Abstract

Gas gangrene, caused mainly by the anaerobic bacterium *Clostridium perfringens* (*C. perfringens*), causes death within 48 h of onset. Limited therapeutic strategies are available, and it is associated with extremely high mortality. Both *C. perfringens* alpha toxin (CPA) and perfringolysin O (PFO) are important virulence factors in the development of gas gangrene, suggesting that they are therapeutic targets. Here, we found that verbascoside, a phenylpropanoid glycoside widely distributed in Chinese herbal medicines, could effectively inhibit the biological activity of both CPA and PFO in hemolytic assays. The oligomerization of PFO was remarkably inhibited by verbascoside. Although no antibacterial activity was observed, verbascoside treatment protected Caco-2 cells from the damage caused by CPA and PFO. Additionally, infected mice treated with verbascoside showed significantly alleviated damage, reduced bacterial burden, and decreased mortality. In summary, verbascoside has an effective therapeutic effect against *C. perfringens* virulence both *in vitro* and *in vivo* by simultaneously targeting CPA and PFO. Our results provide a promising strategy and a potential lead compound for *C. perfringens* infections, especially gas gangrene.

## Introduction

*Clostridium perfringens* (*C. perfringens*) is a gram-positive anaerobic bacterium that is widely distributed in nature and in the human intestine (Fraser and Collee, 1975). *C. perfringens* can secrete four major toxins (α, β, ε, and) and is accordingly divided into five serotypes, A–E (Titball, 2005). In humans, *C. perfringens* can cause a variety of diseases, such as food poisoning and gas gangrene, the latter of which can also occur in animals, such as cats, cattle, dogs, sheep, horses, and goats (Verherstraeten et al., 2015). The incubation period of gas gangrene is short, and the infection can become established within 6–8 h (Uzal et al., 2015). More seriously, more than 50% of those infected will continue to develop systemic toxemia, eventually leading to death; thus, without the timely and effective administration of treatment, gas gangrene can lead to serious economic losses (Low et al., 2018). However, the usual treatments, such as antibiotic treatment and surgical treatment for gas gangrene, all have certain disadvantages including but not limited to antibiotic resistance and amputation injury to patients (Park et al., 2010; Osman and Elhariri, 2013). Thus, it is necessary to find new methods or ideas for the treatment of gas gangrene.

Clostridial gas gangrene is the fulminant infectious disease caused mainly by the interaction of α *C. perfringens* toxin (CPA) and perfringolysin O (PFO) (Hifumi et al., 2018). CPA, belonging to the family of bacterial zinc-metallo phospholipase enzymes, is produced by all *C. perfringens*, is encoded by chromosomes, is a major toxin in *C. perfringens*, and exhibits both phospholipase C and sphingomyelinase activities (van Bunderen et al., 2010; Masataka et al., 2015). In the 1990s, Awad et al. (1995) used CPA mutants to test a gas gangrene mouse model and found that strains lacking the CPA gene showed decreased pathogenicity and less tissue damage after infection. This work confirmed that CPA is the major toxin in gas gangrene. In 2000, Alberto conducted in-depth studies by constructing a molecular model of α-toxin and three loop mutants in the C-terminal domain, and the hemolytic activity and cytotoxicity of the mutants were decreased compared to those of the wild type. It was demonstrated that sphingomyelinase activity and the C-terminal domain are necessary for myotoxicity *in vivo* (Alape-Giron et al., 2000). PFO, a cholesterol-dependent cytolysin (CDC), interacts with the cell membrane as a monomer, and the prepore complex is then inserted into the membrane bilayer by oligomerization, finally lysing host cells (Johnson and Heuck, 2014). Although PFO is not necessary for lethality, it can cause macrophage cytotoxicity in the early stages of myonecrosis and is important for thrombus formation in the late stages of infection, contributing to the pathogenesis of gas gangrene (Verherstraeten et al., 2015). CPA can combine with PFO to damage neutrophils accumulated at the infected site and induce endothelial cell dysfunction, edema, and ischemia, finally leading to tissue hypoxia and providing a favorable anaerobic environment for the growth and reproduction of *C. perfringens* (Bryant and Stevens, 1996). Thus, this study targets CPA and PFO and aims to reveal a new method for treating gas gangrene.

In this study, two toxin inhibitors were screened from 17 natural compounds by hemolysis to find a potential inhibitor of gas gangrene. The hemolytic data for various compounds are shown in Table 1. Among the 17 kinds of natural compounds, verbascoside has the best inhibitory effect on both toxins, and we thus selected verbascoside as the target compound for this study. Verbascoside is widely distributed in various Chinese herbal medicines and was isolated and extracted from *Verbascum sinuatum* by an Italian scientist in 1963 (Speranza et al., 2010; Zhu et al., 2016). Studies have shown that verbascoside has many biological and pharmacological effects, such as anti-inflammatory, antioxidant, antibacterial, antitumor, antifungal, and chelation properties, and can be used in skin cosmetics and topical preparations (Mazzon et al., 2009; Kostyuk et al., 2011; Vertuani et al., 2011; Etemad et al., 2016). Other reports have shown that verbascoside can be fully absorbed within 1 h and reach a high blood concentration and that its bioavailability on delivery via various injection methods does not exceed 25% (Dai et al., 2017; Feng et al., 2018). Based on the aforementioned pharmacological activities, this study found and studied the inhibitory effects of verbascoside on gas gangrene through a series of *in vitro* and *in vivo* experiments.

**TABLE 1:** Natural compounds tested in this study.

**Serial number**	**Natural compound**	**Hemolytic activity (%)**	
		**PFO**	**CPA**
1	Verbascoside	7.17	5.54
2	Silymarin	31.88	14.07
3	Aloeemodin	108.20	98.51
4	Phloretin	76.69	96.03
5	Baicalein	82.86	6.36
6	Albiflorin	90.14	96.13
7	Loganin	92.09	91.69
8	Baicalin	74.88	92.54
9	Astragaloside A	80.01	94.70
10	Luteolin	69.38	89.52
11	Alantolactone	73.92	81.77
12	Myricetin	29.48	4.41
13	Chalcone	79.79	76.59
14	Morin hydrate	45.70	79.14
15	Rosmarinic acid	59.70	95.55
16	Asiatic acid	19.28	2.84
17	Piperine	102.44	81.96
18	Positive control	100.00	100.00
19	Negative control	0.00	0.00

*The concentration of each compound used in this study was 32 μg/mL.*

## Materials and Methods

### Bacterial Growth and Reagents

ATCC13124 served as the *C. perfringens* strain used in this study. Brain heart infusion (BHI) and tryptone soy broth (TSB) were purchased from Qingdao Hope Biol-Technology Co., Ltd. (Qingdao, China). All the tests for *C. perfringens* in this study were conducted in the P2 laboratory.

*Escherichia coli* BL21(DE3) containing the PET-28a-PFO plasmid was stored in our laboratory. The protein phospholipase C (CPA) was purchased from Sigma Aldrich, Shanghai, China.

Verbascoside (purity >98%) was purchased from Chengdu Desite Biotech Co., Ltd. (Chengdu, Sichuan, China). Isopropyl β-D-1-thiogalactopyranoside (IPTG) was purchased from Dalian Meilun Biotechnology Co., Ltd. (Dalian, China).

### Preparation and Purification of PFO

The gene encoding PFO was amplified using primers containing *Bam*HI and *Xho*I digestion sites, ligated into the PET-28a plasmid, and transformed into DH5α competent cells. After sequencing, the plasmid was extracted, transformed into BL21(DE3) competent cells, and stored at −20°C for the following assays. BL21 (DE3) cells carrying the PET-28a-PFO plasmid were cultured (37°C, 200 rpm) in TSB containing kanamycin until reaching an OD_600_ nm of 0.6–0.8. IPTG was added to the cells at a final concentration of 0.5 mM, and the cells were cultured at 16°C for 12 h at 200 rpm. The cells were then centrifuged and resuspended in phosphate-buffered saline (PBS) buffer (pH = 7.4). Following sonication (20 HZ, 100 W) and centrifugation (10,000 rpm, 1 h), the supernatant was collected and passed through a His-affinity column (GE Amersham, Shanghai, China). The PFO samples were eluted with 50 mM imidazole, concentrated in PBS buffer (pH = 7.4), and stored at −20°C for the following study. The purchased CPA powder (Sigma Aldrich) was dissolved in PBS buffer and stored at −20°C for further study. The primers used in this study were as follows: F: 5-GCGCGGATCCATGGCTTTATGTCTGTTT-3 and R: 5-GCGCCTCGAGTTAATTGTAAGTAATACTAG-3.

### Hemolytic Activity Assay

First, 2 μL of PFO (8 μg) or CPA (3.2 μg) protein was added to 485.5 μL of PBS buffer supplemented with various concentrations of verbascoside (0, 2, 4, 8, 16, 32 μg/mL) and preincubated at 37°C for 20 min. Then, 12.5 μL of rabbit or sheep red blood cells (2.5%) was added to each group to a final volume of 500 μL, and the mixture was incubated at 37°C for 20 min. After centrifugation (12,000 *g*, 1 min, 4°C), the supernatant of each sample was collected, and the absorbance was measured at OD_543_ nm. The sample without protein and verbascoside was used as a negative control, and the sample treated with H_2_O was used as a positive control.

### Minimal Inhibitory Concentration Determination Assay

The minimal inhibitory concentration (MIC) value of verbascoside against *C. perfringens* ATCC13124 was determined by the agar dilution method according to a previous study (Beshiru et al., 2019). Briefly, *C. perfringens* ATCC13124 was adjusted to 1 × 10^5^ colony-forming units (CFUs), added to the surface of each blood plate, placed in an anaerobic bag, and incubated at 37°C for 48 h.

### Bacterial Growth Curve Assay

*Clostridium perfringens* ATCC13124 was inoculated into 50 mL of BHI and cultured at 37°C in a 2.5-L anaerobic culture bag until reaching an OD_600_ nm value of 0.3. Then, the bacteria were aliquoted and cocultured with different concentrations of verbascoside (0, 4, 8, 16, 32, 64, or 128 μg/mL). Finally, the bacteria were cultured at 37 C in a 2.5-L anaerobic culture bag. Cell growth was determined every hour by measuring each group of bacteria at OD_600_ nm.

### Lactate Dehydrogenase Assay

Caco-2 cells (ATCC HTB-37^TM^) were plated into a 96-well plate at 2.5 × 10^4^ cells per well and placed in a 5% CO_2_ incubator for 24 h. Then, verbascoside was diluted to different concentrations (0.25–2 μg/mL) with RPMI 1640 cell culture medium without serum or antibiotics and added to each well. Finally, PFO (3.8 μg) or PLC (2.4 μg) was added to a final volume of 200 μL in each well. The samples were treated with 0.2% Triton X-100 or RPMI 1640 medium as the positive (A^+^) and negative (A^–^) controls, respectively. The other samples were treated with protein and the indicated concentration of verbascoside (A). After 5 h of incubation in 5% CO_2_, the plates were centrifuged at 1,000 rpm for 10 min. The absorbance of the supernatant in each well was measured at a wavelength of 492 nm by adding a cytotoxicity test reagent (LDH; Roche, Basel, Switzerland). The cytotoxicity was calculated according to the following formula: (A−A^–^)/(A^+^−A^–^) × 100%.

### Live/Dead Cell Assay

Cells were plated in a 96-well plate overnight at a density of 2.5 × 10^4^ cells per well. The protein (PFO 3.8 μg or PLC 2.4 μg), premixed with various concentrations of verbascoside, was added to each well and incubated for 5 h at 5% CO_2_. Then, the protective effects of verbascoside on the cells were assessed by using the live/dead (green/red) reagents (Roche) to observe under a fluorescence microscope according to the manufacturer’s instructions.

### Oligomerization Assay

First, 5 μL of purified PFO (15 μg) protein was added to PBS buffer with different concentrations of verbascoside (0, 8, or 32 μg/mL) and incubated at 37°C for 20 min. Then, 100 μL of 428 mM KCl was added, and the solution was mixed and incubated at 37°C for 10 min. Finally, rabbit red blood cells were added to the system to make the final concentration 0.5%, and the oligomerization reaction of PFO was induced. The total volume of the system was 500 μL. Next, 5× loading buffer without β-mercaptoethanol was added, and the solution was heated at 55°C for 10 min. The formation of PFO oligomers and monomers was monitored by Western blot as described previously (Wang et al., 2015) using the rabbit anti-perfringolysin O antibody (primary antibody; Abcam, Cambridge, United Kingdom) and peroxidase-conjugated antibody (secondary antibody; Proteintech, Beijing, China). His-tagged *Coxiella burnetii* effecter protein (30 KD) was used as the loading control and detected with a His-tagged antibody.

### *In vivo* Experiments

Female BALB/c mice (6–8 weeks old) were purchased from Liaoning Changsheng Biotechnology Co., Ltd. (Changchun, China) and the experiments were approved by and conducted in accordance with the guidelines of the Animal Care and Use Committee of Jilin University.

*Clostridium perfringens* ATCC13124 was inoculated into BHI in an anaerobic culture bag at 37°C until reaching an OD_600_ nm value of 1.5, followed by centrifugation at 5,000 rpm for 5 min to collect the bacteria. Then, the bacteria were resuspended in PBS buffer (pH = 7.4) for further study. In addition, each mouse was subcutaneously administered 100 mg/kg verbascoside after the infection and at 8-h intervals until a predefined time point was reached.

A total of 2 × 10^8^ CFUs of *C. perfringens* ATCC 13124 was intramuscularly injected into mouse legs. The infected mice were treated with verbascoside as described earlier or with DMSO on the same schedule. The dead mice were collected at each time point in each group within 72 h to analyze mortality, and 15 mice were used in each group. For other studies (12 mice per group), 2 × 10^7^ CFUs bacteria were infected into mouse leg muscles by the same treatment method with verbascoside or DMSO as described earlier. At 48 h post-infection, the mice were sacrificed by cervical dislocation and necropsied. For pathologic examination, the leg muscles were placed in 4% paraformaldehyde, stained with hematoxylin and eosin, and observed under a light microscope. To assess the effect of verbascoside on the bacterial burden of the leg muscles, the muscle of the infected part of the leg was removed, weighed, homogenized in PBS, diluted, and inoculated onto BHI solid agar plates for 18 h at 37°C.

### Statistical Analysis

All assay data analyses (*n* ≥ 3) were performed using SPSS 19.0 software, and *P*-values were calculated using one-way analysis of variance (ANOVA; ^∗^*P* < 0.05 and ^∗∗^*P* < 0.01). The survival data were analyzed using the log rank test (^∗^*P* < 0.05 and ^∗∗^*P* < 0.01).

## Results

### Verbascoside Inhibited the Hemolytic Activity of PFO and CPA

**FIGURE 1:**
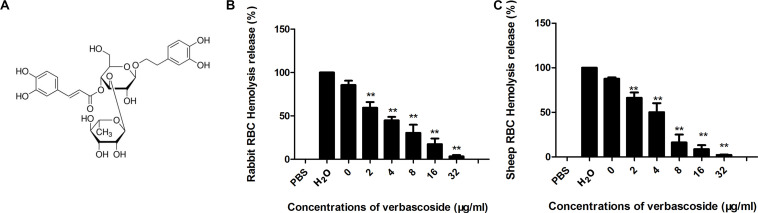
Verbascoside inhibits CPA and PFO hemolytic activity. **(A)** Chemical structure of verbascoside. **(B,C)** Hemolytic assays were performed by the coincubation of PFO and rabbit red blood cells or CPA and sheep red blood cells in PBS buffer. The hemolytic activity of each sample was determined by comparing the OD_543_ nm values of the supernatant sample and control sample treated with H_2_O (without verbascoside, used as 100%). The data are shown as the mean ± SD values of three independent experiments. *P*-values were calculated using one-way analysis of variance (ANOVA) (**P < 0.01).

Some flavonoids have been reported to be effective inhibitors against pore-forming toxins (Li et al., 2013; Wang et al., 2017; Zhou et al., 2017). Here, verbascoside (Figure 1A), a phenylpropanoid, was found to inhibit the hemolytic activity of both the PFO and CPA toxins secreted by *C. perfringens*. As shown in Figures 1B,C, in the positive control, the RBCs could be completely lysed by H_2_O with 100% hemolysis activity. The sample treated with CPA or PFO without verbascoside was used as the 0 μg/mL. When the concentrations of verbascoside ranged from 2 to 32 μg/mL, verbascoside treatment significantly inhibited the hemolytic activity of these toxins in a dose-dependent manner, indicating that verbascoside is an effective inhibitor of both PFO and CPA.

### Verbascoside Does Not Affect the Growth of *C. perfringens*

**FIGURE 2:**
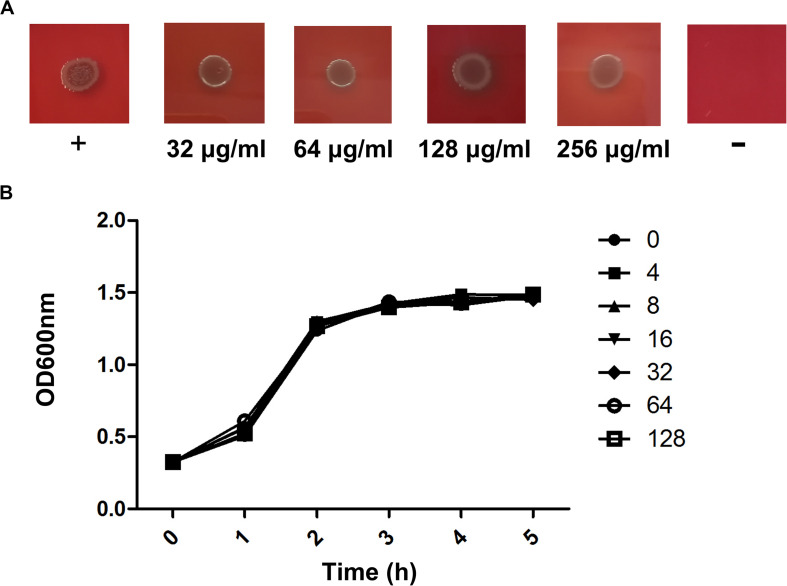
Verbascoside has no influence on the growth of *C. perfringens*. **(A)** The MIC value of verbascoside against *C. perfringens* ATCC13124 was determined by agar dilution. **(B)** Growth curves for the *C. perfringens* strain ATCC13124 administered different concentrations of verbascoside were examined by monitoring the OD600 nm of each sample every 60 min.

We further performed MIC and bacterial growth curve studies to determine the antibacterial activity of verbascoside against *C. perfringens*, revealing an MIC assay result (Figure 2A) >256 μg/mL. Similarly, the bacterial growth curve results (Figure 2B) indicate that verbascoside does not affect the normal growth of the bacteria. Together, our results indicate that verbascoside treatment has no influence on *C. perfringens* viability at the concentrations required for the inhibition of PFO and CPA activity.

### Verbascoside Protects Cells From Damage Induced by PFO or CPA

**FIGURE 3:**
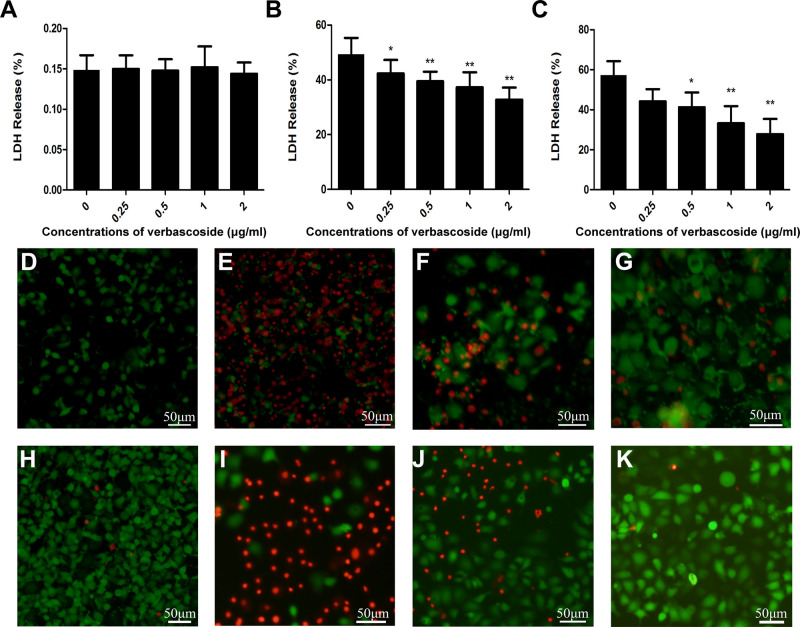
Verbascoside protected Caco-2 cells from injury induced by PFO or CPA. **(A)** Verbascoside was incubated with Caco-2 cells for 5 h, and cytotoxicity was detected by a cytotoxicity detection kit. Verbascoside reduced the cell damage caused by **(B)** PFO and **(C)** CPA. Caco-2 cells were cocultured with verbascoside preincubated with PFO **(B)** or CPA **(C)** for 5 h, and cytotoxicity was detected by a cytotoxicity detection kit. The data are shown as the mean ± SD values of three independent experiments. *P* values were calculated using one-way analysis of variance (ANOVA; *P < 0.05 and **P < 0.01). Caco-2 cells were cocultured with verbascoside preincubated with **(D–G)** PFO or **(H–K)** CPA for 5 h and observed under fluorescence microscope using the live/dead (green/red) reagent. Green and red represent live and dead cells, respectively. Scale bar, 50 μm.

*Clostridium perfringens* alpha toxin and PFO in *C. perfringens* are the main toxins that cause gas gangrene; thus, whether verbascoside could protect cells from these two toxins was also examined. First, we carried out a cytotoxicity test with the drug verbascoside, which did not damage the cells (Figure 3A). Then, this compound was added into the coculture system of cells and PFO or CPA. As shown in Figures 3B,C, verbascoside inhibited the cytotoxicity of these two toxins in Caco-2 cells. Compared with samples not treated with verbascoside, the damage induced by the toxins to Caco-2 cells treated with verbascoside at 0.5 μg/mL was significantly reduced in a dose-dependent manner. Especially for the PFO protein, the cells were effectively protected at a drug concentration of 0.25 μg/mL (*P* < 0.05). A subsequent cell viability/death assay (Figures 3D–K) was further employed to prove this protective effect. As expected, verbascoside treatment visibly protected against cell injury induced by PFO or CPA. Taken together, our results established that verbascoside significantly inhibits the cell damage induced by PFO or CPA in a dose-dependent manner.

### Verbascoside Inhibits PFO Hemolytic Activity by Decreasing Its Oligomerization

**FIGURE 4:**
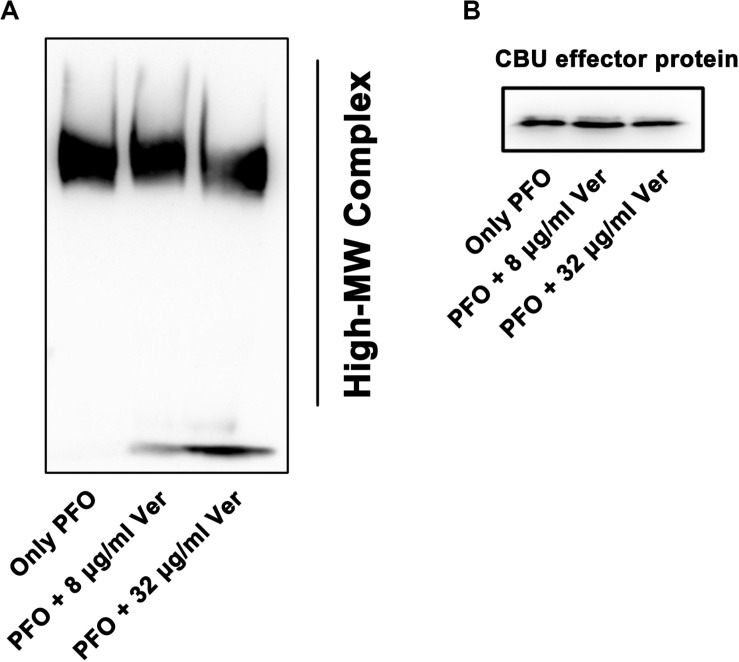
Verbascoside prevents oligomerization of PFO. **(A)** Oligomerization of PFO pretreated with different concentrations of verbascoside was induced by KCl and rabbit red blood cells in PBS buffer. The formation of oligomers was detected by Western blotting. **(B)** His-tagged *Coxiella burnetii* effector protein (30 KD) was used as the loading control and detected with a His-tagged antibody.

The hemolytic activity of PFO must be accompanied by the occurrence of oligomerization and pore formation (Johnson and Heuck, 2014). Here, the oligomerization of PFO was induced by KCl and rabbit blood, and PFO oligomerization was detected (Figure 4), revealing a decreased oligomerization band intensity when the concentration of verbascoside was increased. Together, our results suggest that verbascoside treatment inhibits the oligomerization of PFO and thereby reduces the hemolytic activity of this toxin.

### Verbascoside Protects Mice From *C. perfringens* Infection

**FIGURE 5:**
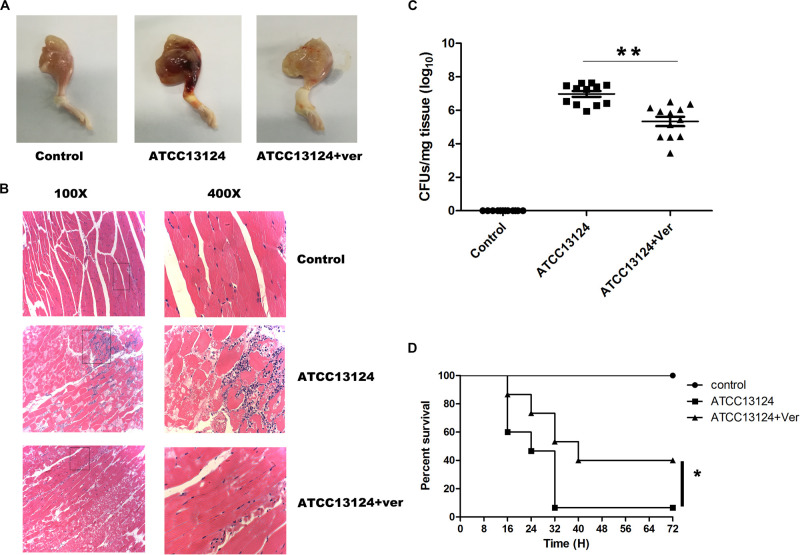
Verbascoside protected mice from *C. perfringens* gas gangrene. **(A)** Pathologic photographs and **(B)** tissue sections of the leg tissues of mice administered the indicated treatment, in which the tissue section magnifications are 100× (eyepiece 10×, objective lens 10×) and 400× (eyepiece 10×, objective lens 40×). **(C)** The bacterial load of the leg muscles was measured 48 h after infection using a plated assay. Three independent experimental results were subjected to statistical analysis (*P < 0.05 and **P < 0.01). **(D)** After injection of 2 × 10^8^ CFUs of *C. perfringens*, treatment with DMSO or verbascoside was performed, and the survival rate was recorded every 8 h for 72 h.

A mouse leg muscle model of gangrene was established to determine whether the protection observed *in vitro* could also be observed *in vivo*. The legs of ATCC13124-infected mice were markedly red and swollen, accompanied by extensive hyperemia and hemorrhagic clots (Figure 5A). In line with the foregoing observation, verbascoside treatment significantly alleviated such damage, with only a small bleeding point and swelling, which were almost normal compared to those in control mice without infection (Figure 5A). A light microscope revealed that the muscle tissue was seriously damaged, and the inflammatory cells were concentrated in the muscle tissue space in the sample infected with ATCC 13124 (Figure 5B). In contrast, the number of inflammatory cells in the drug group was significantly reduced, and the tissue was intact following treatment with verbascoside (Figure 5B). In addition, the number of bacteria colonized in the leg muscles was significantly reduced after treatment with verbascoside (Figure 5C). As shown in Figure 5D, 93.33% of infected mice died by 32 h post-infection. As expected, the survival rate of mice receiving verbascoside increased by 33.33%, as only 60% of the infected mice died (*P* = 0.015 by a log rank test). Furthermore, verbascoside treatment delayed the death period peak by nearly 8 h (from 32 h for ATCC 13124-infected mice to 40 h for verbascoside-treated mice) and could thus extend the precious time required for clinical rescue (Figure 5D). Taken together, our results indicated that in mice, verbascoside can provide systemically effective protection against gas gangrene caused by *C. perfringens*.

## Discussion

As a gram-positive anaerobic bacterium, *C. perfringens* is widely distributed in nature and can secrete a variety of toxins, which can lead to a variety of diseases in humans and animals, including gas gangrene (Fraser and Collee, 1975; Verherstraeten et al., 2015). Gas gangrene is mainly caused by the combination of α and theta toxins secreted by *C. perfringens*, which can lead to the death of infected animals, resulting in great economic losses. However, to date, effective methods for treating gas gangrene include hyperbaric oxygen, surgery, and antibiotics. Among them, surgery can cause substantial physical damage to patients and have a very inconvenient impact on postoperative life. Numerous tetracycline resistance genes have been found in *C. perfringens* isolates from Sweden, Norway, Denmark, and other places. Thus, novel strategies or agents are needed to fight this bacterial infection (Park et al., 2010; Osman and Elhariri, 2013). In this study, the phenylpropanoid verbascoside, which differs from traditional antibacterial treatments (antibiotics), could inhibit the hemolytic activity of both CPA and PFO, two important pore-forming toxins that critically contribute to *C. perfringens* pathogenicity. At the cellular level, verbascoside could also effectively protect Caco-2 cells from these two toxins at low concentrations (0.25–2 μg/mL). In particular, verbascoside could reduce the activities of the toxins by inhibiting the oligomerization of PFO. These results could provide sufficient basic data for *in vivo* research.

Cases of deep injury or improper postoperative care can cause gas gangrene, especially after natural disasters, such as an earthquake. Poor working environments lead to the easy contraction of gas gangrene, and the time between disease onset and death is typically only 48 h (Wang et al., 2010; Chen et al., 2011). Thus, missing the golden treatment period can have very serious consequences. The animal experimental results in this study demonstrated that verbascoside could remarkably reduce the pathogenic damage of *C. perfringens* gas gangrene (leg muscle) and significantly decrease the colonization of *C. perfringens* in the leg muscles. Furthermore, the survival rate of the infected mice increased by 33.33% following verbascoside treatment, and verbascoside also delayed the outbreak of death, which is ideal for reaching the effective gold standard rescue time for the treatment of gas gangrene. Currently, only hyperbaric oxygen therapy, amputation surgery, and antibiotics are available for the treatment of gas gangrene (Slack, 1976; Anjana et al., 2014; Ryohei et al., 2018), but the aforementioned methods damage the affected body region and do not prolong the effective treatment time. Herein, verbascoside treatment was shown to prolong the treatment time for gas gangrene without causing serious bodily damage, which fills the gap in the treatment of this infection. In addition, PFO can combine with CPA to damage host cells, which eventually leads to tissue hypoxia and benefits the growth and reproduction of *C. perfringens* in anaerobic environments. Although verbascoside exhibited no antibacterial activity against *C. perfringens*, it could simultaneously inhibit the activities of these two toxins in this study. Owing to this inhibitory effect, verbascoside treatment interfered with the establishment of infection by *C. perfringens* and subsequently inhibited the bacterial burden in *C. perfringens*-infected mice. These data showed that, unlike antibiotics, verbascoside treatment may not put survival pressure on *C. perfringens*.

Our laboratory previously found that many Chinese herbal extracts can inhibit the hemolytic activity of pore-forming toxins secreted by bacteria, including *Staphylococcus aureus*, *Listeria monocytogenes*, and *Streptococcus pneumoniae* (Liu et al., 2017; Song et al., 2017; Teng et al., 2017). For example, luteolin can affect the infection of *L. monocytogenes* by inhibiting the production of LLO. Additionally, verbascoside was also identified as an effective inhibitor of *S. pneumoniae* PLY by directly neutralizing its activity (Zhao et al., 2016). Interestingly, the PLY and PFO described in this study are two important toxins in the CDC family. Owing to the high structural homology of the CDC family, verbascoside may inhibit the activities of other CDCs, such as SLY and LLO. Therefore, verbascoside may be used as a potential candidate against infections of bacteria that produce CDCs. However, after 50 mg/mL verbascoside was intramuscularly injected into rats, the *C*_max_ in mice was approximately 20,000 ng/mL (Feng et al., 2018), which indicated that the bioavailability of this compound was relatively low. Thus, further studies should be performed to improve the bioavailability of verbascoside.

In summary, we offer a new strategy for the treatment of gas gangrene by inhibiting CPA and PFO. This strategy also plays a role in the treatment of other pathogenic infections, indicating that verbascoside is a potential lead compound for the treatment of gas gangrene.

## Data Availability Statement

The raw data supporting the conclusions of this article will be made available by the authors, without undue reservation, to any qualified researcher.

## Ethics Statement

The animal study was reviewed and approved by the Animal Care and Use Committee of Jilin University.

## Author Contributions

JW, JH, and JZ conceived and designed the experiments. JZ, SL, LX, ZW, NH, and TW performed the experiments. XD contributed reagents, materials, and analysis tools. JW, JH, and JZ wrote the manuscript. All authors contributed to the article and approved the submitted version.

## Conflict of Interest

The authors declare that the research was conducted in the absence of any commercial or financial relationships that could be construed as a potential conflict of interest.
